# Malignant Carotid Paraganglioma: A Case Report

**DOI:** 10.7759/cureus.41765

**Published:** 2023-07-12

**Authors:** Maani M Archang, Seung Lee, Ismail Ziu, William Clifton, David A Miller, Mark E Jentoft, Jeffrey R Janus

**Affiliations:** 1 Otolaryngology - Head and Neck Surgery, University of California, Los Angeles, Los Angeles, USA; 2 Neurological Surgery, Mayo Clinic, Jacksonville, USA; 3 Neurosurgery, Ascension Medical Group St Vincent's Spine & Brain, Jacksonville, USA; 4 Center for Spine Health, Cleveland Clinic, Cleveland, USA; 5 Radiology, Mayo Clinic, Jacksonville, USA; 6 Laboratory Medicine and Pathology, Mayo Clinic, Jacksonville, USA; 7 Otolaryngology - Head and Neck Surgery, Mayo Clinic, Jacksonville, USA

**Keywords:** carotid body tumor, internal carotid artery ligation, malignant paraganglioma, presurgical embolization, carotid paraganglioma

## Abstract

Carotid body tumors (CBTs) are rare neoplasms of the neuroectoderm accounting for 0.6% of head and neck tumors, with a 2%-12.5% risk of malignancy. While surgical resection has been associated with a high rate of neurologic and vascular complications, it remains the mainstay of treatment for malignant CBTs. We present the case of a 40-year-old female with a 5-year history of progressively enlarging right-sided neck mass, with MRI and MRA showing a Shamblin grade III CBT encasement of the internal carotid artery (ICA). Blood flow was absent in the petrous segment of ICA, with great collateralization of brain blood supply, enabling en bloc resection of the tumor with a carotid bulb and ligation of the common carotid artery (CCA) without vascular reconstruction. Further, we describe the characteristics and current management for malignant CBTs, including surgical management, pre-surgical embolization, and adjuvant radiation therapy.

## Introduction

Carotid body tumors (CBTs) are rare neoplasms of the neuroectoderm arising from a gangliocytoma at the carotid bifurcation, accounting for 0.6% of head and neck tumors, with a 2-12.5% risk of malignancy [1-2]. CBTs are usually benign and more frequently diagnosed in elderly patients, 50-70 years of age. In contrast, malignant CBTs are more commonly present at 20-50 years of age [3].

Malignant CBTs most commonly present with a slowly growing firm neck mass without other symptoms, but compared to benign CBTs, they are more likely to be symptomatic presenting as a painless, mobile, pulsatile mass. Infiltration of cranial nerves IX, X, XI, and XII and the carotid artery can lead to hoarseness, ear pain, dysphagia, dizziness, headache or orbital pain, stridor, and Horner's Syndrome [3]. Bilateral and symptomatic tumors are more likely to be malignant. Vascular phenomena such as pulsations or murmurs, cerebral ischemia or carotid sinus syndrome with bradycardia and syncope may occur with infiltration or compression of the common carotid artery.

The mechanism of developing carotid body paragangliomas is not fully understood. However, chronic hypoxia in cases such as cardiopulmonary disease, living at high altitude, or disruption of oxidative phosphorylation such as mutations in the mitochondrial succinate dehydrogenase complex (SDH) has been suggested as an underlying cause of carotid body hypertrophy leading to CBT [1, 4].

The initial diagnosis of CBTs is based on imaging findings showing a low-resistance blood flow pattern. Doppler ultrasound and CT/MRI are mainstays of imaging. CT and MRI are methods of choice for assessment of size and degree of invasiveness. CT or MRI angiography is the gold standard for diagnosing tumor relation with vessels. Shamblin classification, which indicates the extension of the tumor around the great vessels of the neck, does not correlate with malignancy, however, it is an indicator of intra-operative and post-op complications.

There is no consensus on histopathological markers of CBT malignancy. Instead, the definitive diagnosis of malignant CBTs is established with the regional invasion of lymph nodes, blood vessels, and perineural space, as well as skull base structures or distant metastasis in tissues where chromaffin cells are normally absent [5]. Furthermore, sporadic cases of CBT have a higher rate of malignancy than familial cases, with the exception of succinate dehydrogenase complex iron sulfur subunit B (SDHB) mutation which carriers a 19-fold or greater odds of malignancy [4, 6]. As such SDHB mutation testing is recommended in all cases of head and neck paragangliomas. CBTs present rarely with RET, VHL, and NF1 mutations, and genetic testing in these genes is not routinely recommended unless family history is suggestive of MEN2, VHL, or NF1 [5]. Genetic testing should also be offered to healthy first-degree relatives of patients with suspected or confirmed hereditary paragangliomas and second-degree relatives in the case of SDHD and SDHAF2 mutations.

Complete surgical resection, unless contraindicated, is the gold standard for the treatment of paragangliomas and can be combined with pre-embolization and adjuvant radiotherapy. Watchful waiting and radiation therapy, with or without conservative surgery are also possible treatment pathways based on clinical context and goals of care. We will cover treatment options in more detail in the discussion section. Malignant CBTs of the neck follow a more indolent clinical course compared to paragangliomas of other sites with higher 5-year survival rates reported from 60% to 85% for nodal involvement. Although distant metastases are exceedingly rare, they are observed more commonly in bone, lung, and liver, and less commonly in pancreas and brain [3, 7-8]. Metastatic malignant CBTs have a reported 5-year survival rate as low as 11% and can recur up to 20 years after resection, emphasizing the importance of both short-term and long-term follow-up [9-10].

## Case presentation

**Figure 1:**
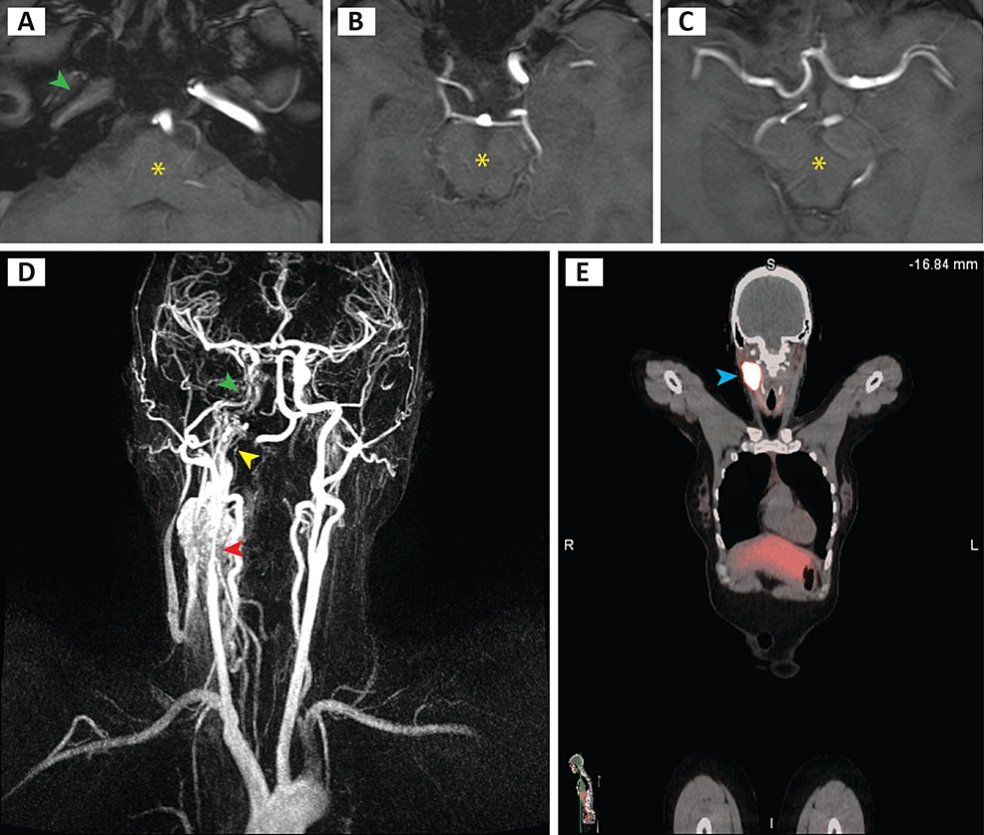
Pre-operative imaging. (A-C) MRI angiography transverse views of the petrous segment and terminal branches of ICA, with yellow asterisk depicting the medulla (A), lower midbrain (B), and upper midbrain (C). D) 3D reconstruction of MRI angiography coronal view. E) PET-CT coronal view. ICA, internal carotid artery; PET-CT, positron emission tomography-computed tomography

A 40-year-old female presented with a 5-year history of progressively enlarging right neck mass. Her previous workup from an outside institution with fine needle aspiration biopsy was inconclusive, and MRI and magnetic resonance angiography (MRA) of the neck revealed a 3.2 cm x 3 cm x 5.5 cm mass encasing the right common carotid artery and proximal internal and external carotid arteries (Figure 1) with severe narrowing of the origin of the right internal carotid artery (ICA) (red arrowhead), absent flow in the right petrous segment (yellow arrowhead), and some retrograde filling to the supraclinoid segment (green arrowhead). CT scan of the neck showed a mass in the right carotid space with associated jugular vein thrombosis extending to the level of the skull base. In addition to the neck tumor (blue arrowhead), positron emission tomography (PET-CT) showed a suspicious left iliac tracer uptake (not shown). Serum and urine metanephrine and normetanephrine were unremarkable. The decision was made to undergo surgery for resection and histological confirmation of the mass.

Pre-operative embolization

**Figure 2:**
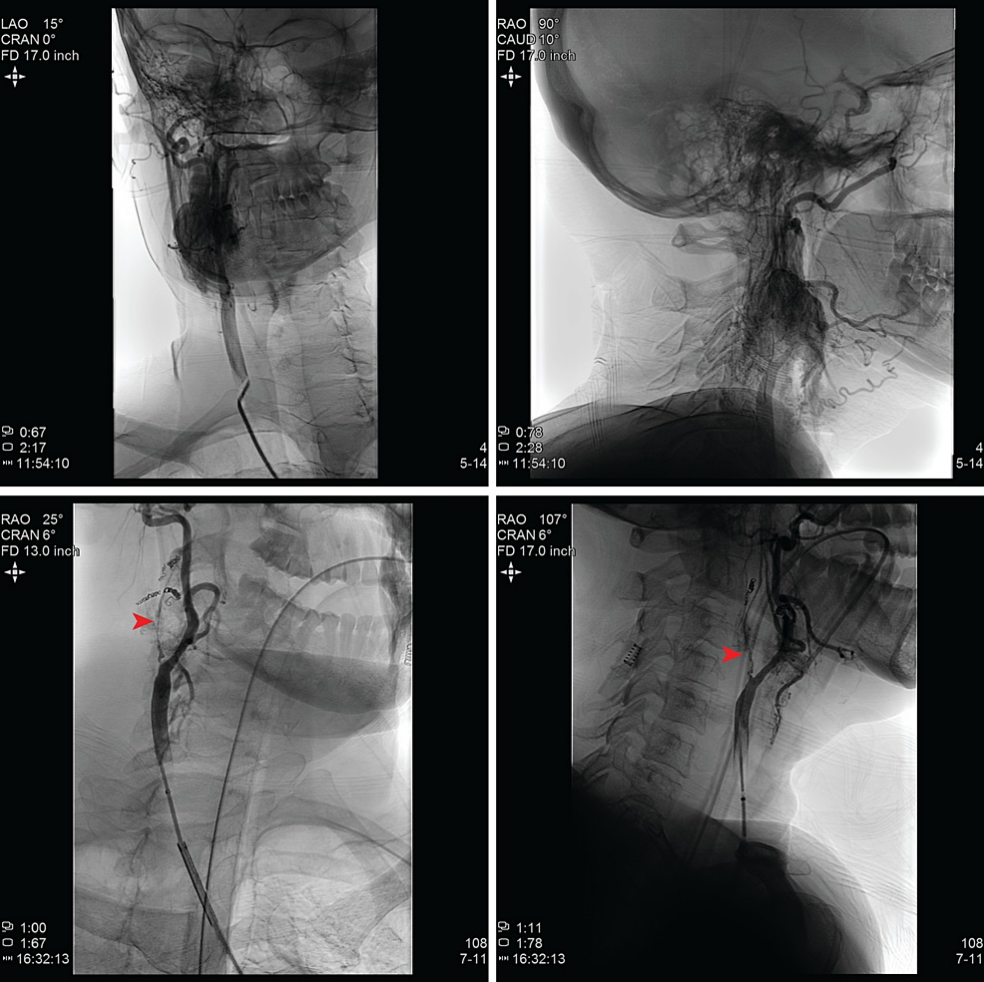
CT angiography during embolization. Rich vascular supply of the tumor (top) and the significant reduction in its blood supply after (bottom) embolization of the feeding arteries.

The patient was taken to the angio-suite for preoperative embolization before the day of surgery. Initial diagnostic angiogram demonstrated absent antegrade flow of the right internal carotid artery (Figure 2 - red arrowhead), and complete occlusion of the right internal jugular vein. Major feeding arteries to the tumor, from the anterior branches of the ascending pharyngeal artery as well as branches of the superior thyroid artery, were identified and embolized (Figure 2 - green arrows). Approximately 75% reduction of the arterial flow to the tumor was achieved.

Operation

**Figure 3:**
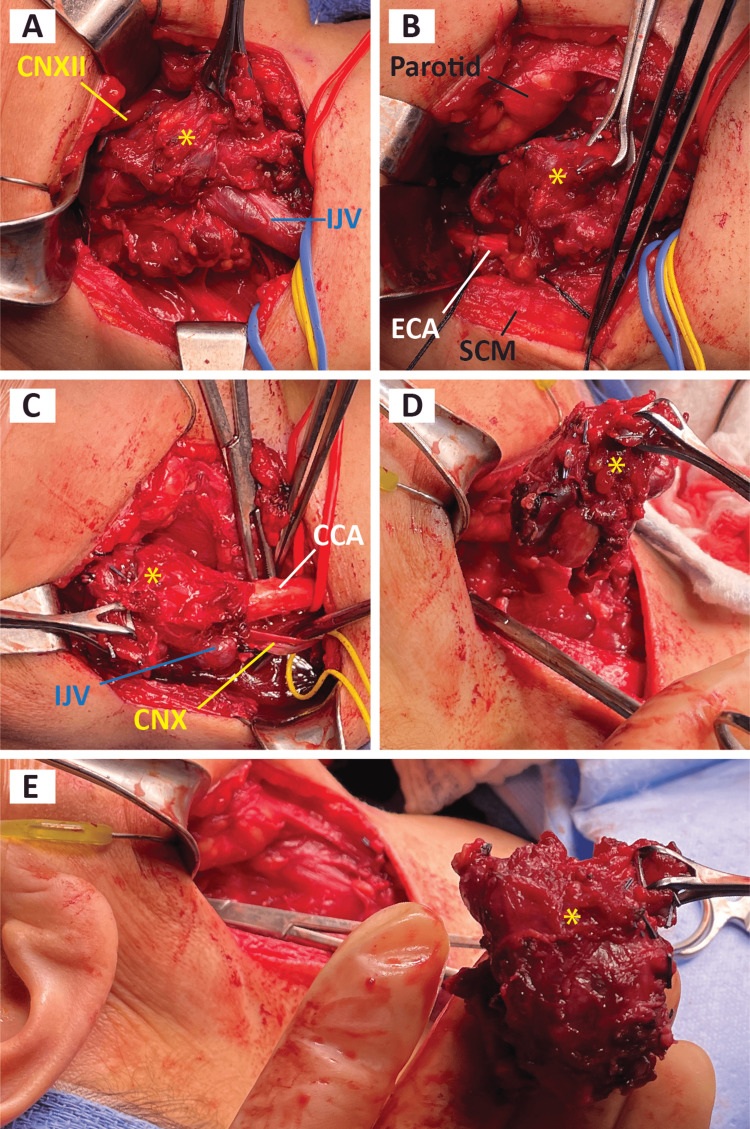
Operative photographs in chronologic order. Orientation: Left is cephalad, up in anterior, Right is caudal. Tumor marked by yellow asterisk. IJV. internal jugular vein; CCA, common carotid artery; ECA, external carotid artery; CNX, vagus nerve; CNXII, hypoglossal nerve; SCM, sternocleidomastoid muscle

The following day, the patient was taken to the operating room for resection of the right-sided neck mass. The incision was made along the neck crease below the angle of the mandible, and subplatysmal flap dissection was carried out down to the carotid sheath inferior to the tumor. After identification and circumferential dissection of the common carotid, jugular vein, and vagus nerve, the dissection was continued superiorly toward the tumor where an invasive and firmly attached mass was wrapping around the carotid structures. The hypoglossal nerve was mobilized safely cephalad to the tumor (Figure 3A). The internal branch of the superior laryngeal nerve was identified and preserved. The external carotid artery superior to the tumor was suture ligated (Figure 3B). The facial artery, lingual artery, ascending pharyngeal artery, and superior thyroid artery were all identified and ligated with clips. With assistance from vascular surgeons, the external carotid artery (ECA) was suture ligated. The CCA was cut and oversewn around the mass invasion into the carotid bulb. The jugular vein was then suture ligated (Figure 3C) and the tumor was mobilized with the carotid bulb and jugular vein (Figure 3D), tracing the vagus nerve up to the grossly enlarged, and not salvageable region. This was then cut with a cuff of normal tissue. The ICA, internal jugular vein (IJV), and vagus nerve were ligated and the tumor was sent to pathology. There was negligible blood loss without intraoperative complications.

The patient had an uneventful postoperative course and was downgraded to the floor from ICU on postoperative day 1. Some hoarseness was noted on the follow-up visit which resolved with a vocal cord injection. The patient was discharged on postoperative day 6. At the 1-month follow-up, the patient was doing well with the recovery of her voice. Pelvis MRI confirmed metastasis over the left ilium and right sacral promontory with additional possible metastasis to the left sacral promontory that was not seen on the PET dota-tate, for which she will receive stereotactic body radiotherapy.

Histopathology

**Figure 4:**
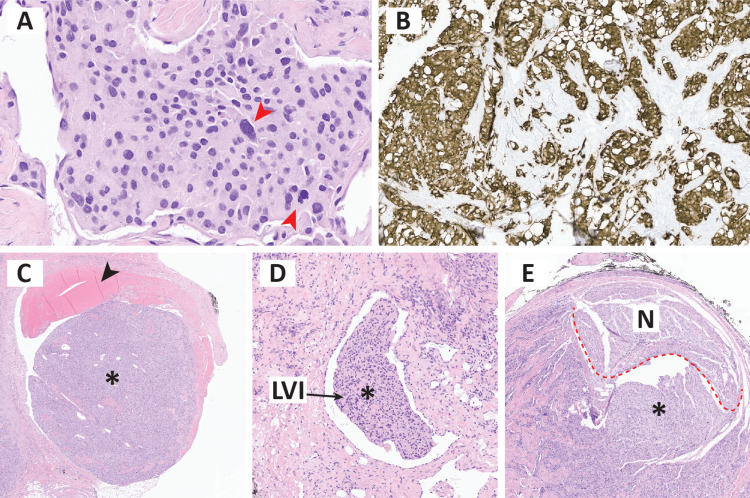
Histopathological assessment of the tumor. A) Synaptophysin immunohistochemistry. B-E) H&E: B) nuclear atypia (red arrowheads), C) blood vessel invasion depicted by presence of tumor (*) in the vascular space containing blood (black arrowhead), D) lymphatic invasion depicted by presence of tumor in vascular space without blood,  E) perineural invasion depicted by presence of tumor (*) adjacent to nerve (above red dashed line).

In histopathological analyses, many tumor cells had round nuclei with stippled chromatin patterns with granular cytoplasm, and in some areas, significant nuclear atypia was seen (Figure 4A). The tissue demonstrated positive staining for synaptophysin (Figure 4B), GATA3, and chromogranin A with S100 highlighting sustentacular cells consistent with paraganglioma. Furthermore, there was an invasion of surrounding blood vessels, lymphatics, as well as a perineural invasion (Figure 4C,E), confirming the malignant nature of the tumor. Further genetic testing for hereditary paraganglioma and pheochromocytoma panel were negative.

## Discussion

Overview of carotid body tumor management

The CBTs are categorized into three classes by Shamblin, et al. [11]. Shamblin I tumors are small and easy to remove. Shamblin II tumors are larger and may involve the great vessels of the neck, but do not encase them. Shamblin III tumors fully encase the vessels and are associated with more complex procedures and neurovascular complications. Most CBTs regardless of malignancy are discovered as Shamblin III.

Surgical removal of CBTs carries a high risk of complications such as the need for vascular reconstruction and transient or permanent nerve damage [3]. As such, watchful waiting, or radiotherapy without resection is reasonable for benign, small, and asymptomatic CBTs, as well as in patients with advanced age, terminal illness, or high anesthetic or surgical risk. The advancing surgical techniques have significantly reduced death from immediate post-op complications, as well as neurovascular complications. As a result, malignancy has surpassed post-resection complications as the leading cause of CBT-related death in patients with CBT. As such, surgical excision is recommended in symptomatic benign CBTs, as well as in cases of malignant or functional paragangliomas [5].

Independent of the Shamblin classification, malignant tumors have a higher chance of intraoperative and post-operative complications. However, the potential for metastatic disease and higher mortality rate in malignant CBTs, make surgery necessary. Primary resection and neck dissection are the mainstay of treatment for malignant paragangliomas. The surgery can be preceded by embolization and followed by adjuvant radiotherapy.

Embolization

There is no consensus on the indication for pre-operative embolization in carotid body paragangliomas. Size, location, and stage have been used as indications. The side effects of embolization make it less commonly performed in Shamblin I and II, vagal, and tympanic paragangliomas. However, it is strongly recommended for large (>3 cm) and extensive tumors such as class C and D jugular paragangliomas and Shamblin class III CBTs [12]. In these tumors, ascending pharyngeal artery, occipital arteries, and branches of the superior thyroid artery, as well as direct blood supplies from carotid arteries may be considered for embolization. The results of embolization may be assessed by MRI with gadolinium contrast (24-48 h after the embolization) where T1W imaging with contrast detects de-vascularized (hypointense) areas. CT scans with iodine contrast (24 h after embolization) give comparable results. A meta-analysis of 22 non-randomized studies has shown patients with embolization had significantly lower blood loss and shorter operative time [13].

Surgical resection and neck dissection

The surgical treatment of choice for malignant or potentially malignant CBTs is resection of the primary tumor and neck dissection [14]. Neck dissection should at least include levels II-IV [15].

Due to proximity to neurovascular structures in the neck, carotid body tumor resection is associated with multiple complications. In a large case series of 229 CBT patients with 16 malignant CBTs, malignant tumors were associated with a higher rate of vascular reconstruction or repair (63% vs. 8%), neurologic complications (69% vs 37%), and significantly longer procedure time [3]. Some 50% of cases with postoperative nerve damage were permanent, with CN XII, sympathetic chain, and CN X being the most affected. In a case-control series of 50 patients with CBTs, complicated cases (bilateral, secretory, non-resectable, or malignant) were associated with a higher rate of nerve complications (45.4% vs. 6.3%) and mortality during follow-up (27.3% vs 0%) [16]. Vascular complications and early mortality were similarly rare in both groups. However, in another larger case series, the rates of vascular reconstruction or repair and neurologic complications were significantly higher in the malignant CBT group. The resection of the carotid body can also result in a decrease in mean arterial pressure [17]. In cases of bilateral CBTs treated with surgery, with resection of the carotid bulb, complete loss of baroreceptor function loss can lead to labile refractory hypertension, headache, and tachycardia.

For rare and challenging malignant tumors which require extensive resection and ligation of the internal or common carotid artery to achieve R0 resection, vascular reconstruction using vascular prostheses or saphenous vein can be used to maintain sufficient blood supply to the brain [18]. Ligation of ICA without reconstruction carries a significant risk of stroke, and death due to the sudden decrease in blood circulation to the ipsilateral brain hemisphere [19-20]. In the case presented here, the right ICA was almost completely occluded, with great collateralization of blood supply to the right side, which enabled en bloc resection of the tumor along with all surrounding vasculature, without compromising the blood supply to the brain.

Adjuvant radiation therapy

Radiotherapy is an effective alternative treatment to surgery for early-stage benign tumors or in patients with extensive tumor or high operative risk and can decrease tumor size or stop growth [21]. However, for functional tumors, radiotherapy alone is not effective, as chief cells secreting catecholamine granules are resistant to radiotherapy [5]. Furthermore, radiotherapy alone for young patients with CBTs has a high risk of malignant conversion and is not recommended. Adjuvant radiation therapy, however, can be used along with resection to reduce recurrence [22].

## Conclusions

Malignant CBTs are rare neoplasms. Surgical resection is the mainstay of their treatment due to the potential for metastatic disease and higher risk of mortality. Extensive vascular involvement in malignant CBTs is challenging and associated with significant intra-operative and post-operative neurovascular complications. Pre-operative embolization of the tumor blood supply can significantly lower blood loss and operative time. In rare cases with severe narrowing of ICA, such as the case presented here, chronic collateralization of cerebral blood flow may enable en bloc resection of the tumor without the need for vascular reconstruction and with excellent postoperative outcome.

## References

[1] Hinojosa CA, Anaya-Ayala JE, Olivares-Cruz S (2018). Malignant shamblin III carotid body tumors resected with use of the retrocarotid dissection technique in 2 patients. Tex Heart Inst J.

[2] Wang YH, Zhu JH, Yang J (2022). The characteristics of carotid body tumors in high-altitude region: analysis from a single center. Vascular.

[3] Zhang W, Liu F, Hou K (2021). Surgical outcomes and factors associated with malignancy in carotid body tumors. J Vasc Surg.

[4] Timmers HJ, Gimenez-Roqueplo AP, Mannelli M (2009). Clinical aspects of SDHx-related pheochromocytoma and paraganglioma. Endocr Relat Cancer.

[5] Berger G, Łukasiewicz A, Grinevych V (2020). Carotid body tumor - radiological imaging and genetic assessment. Pol Przegl Chir.

[6] Neumann HP, Pawlu C, Peczkowska M (2004). Distinct clinical features of paraganglioma syndromes associated with SDHB and SDHD gene mutations. JAMA.

[7] Hu K, Persky MS (2003). Multidisciplinary management of paragangliomas of the head and neck, part 1. Oncology (Williston Park).

[8] Lian L, Liu C, Guan H (2014). [Diagnostic and therapeutic analysis of malignant carotid body tumors]. Zhonghua Yi Xue Za Zhi.

[9] Hamidi O, Young WF Jr, Iñiguez-Ariza NM (2017). Malignant pheochromocytoma and paraganglioma: 272 patients over 55 years. J Clin Endocrinol Metab.

[10] Lee JH, Barich F, Karnell LH (2002). National Cancer Data Base report on malignant paragangliomas of the head and neck. Cancer.

[11] Obholzer RJ, Hornigold R, Connor S (2011). Classification and management of cervical paragangliomas. Ann R Coll Surg Engl.

[12] Boedeker CC (2011). Paragangliomas and paraganglioma syndromes. GMS Curr Top Otorhinolaryngol Head Neck Surg.

[13] Jackson RS, Myhill JA, Padhya TA (2015). The effects of preoperative embolization on carotid body paraganglioma surgery: a systematic review and meta-analysis. Otolaryngol Head Neck Surg.

[14] Hu K, Persky MS (2016). Treatment of head and neck paragangliomas. Cancer Control.

[15] Moskovic DJ, Smolarz JR, Stanley D (2010). Malignant head and neck paragangliomas: is there an optimal treatment strategy?. Head Neck Oncol.

[16] Lozano FS, Muñoz A, de Las Heras JA (2020). Simple and complex carotid paragangliomas. Three decades of experience and literature review. Head Neck.

[17] de Franciscis S, Grande R, Butrico L (2014). Resection of carotid body tumors reduces arterial blood pressure. An underestimated neuroendocrine syndrome. Int J Surg.

[18] Arens C, Granowski D, Udelnow A (2019). Hybrid prosthesis for vascular reconstruction of the internal carotid artery near the skull base after radical excision of a very rare malignant glomus caroticum paraganglioma (article in German). HNO.

[19] Miao B, Lu Y, Pan X (2008). Carotid artery resection and reconstruction with expanded polytetrafluoroethylene for head and neck cancer. Laryngoscope.

[20] Pacheco-Ojeda LA (2017). Carotid body tumors: surgical experience in 215 cases. J Craniomaxillofac Surg.

[21] Dupin C, Lang P, Dessard-Diana B (2014). Treatment of head and neck paragangliomas with external beam radiation therapy. Int J Radiat Oncol Biol Phys.

[22] Williamson J, Leopold G, Prabhu V (2012). Successful treatment of a rare metastatic malignant carotid body tumour in a young adult, with conservative surgery and local radiotherapy. J Laryngol Otol.

